# Suicide mortality in Kazakhstan from 2012 to 2024: temporal trends, demographic inequalities, and regional variation

**DOI:** 10.3389/fpubh.2026.1972530

**Published:** 2026-10-13

**Authors:** Alibek Kossumov, Tolkyn Zhakupova, Nazgul Imambayeva, Tatyana Polyakova, Gulshat Dalibayeva, Bagyzhan Syzdykova, Artur Kovenskiy, Aidana Rakhmankulova, Elizaveta Vinogradova, Sundetgali Kalmakhanov, Samat Kozhakhmetov, Almagul Kushugulova

**Affiliations:** 1 Laboratory of Microbiome, Center for Life Sciences, National Laboratory Astana, Nazarbayev University, Astana, Kazakhstan; 2 Department of Forensic Medicine and Fundamentals of Law, NJSC, Astana Medical University, Astana, Kazakhstan; 3 Center for Forensic Examinations of the Ministry of Justice of the Republic of Kazakhstan, Astana, Kazakhstan; 4 Research Institute of Forensic Examinations of the State Enterprise, Center for Forensic Examinations of the Ministry of Justice of the Republic of Kazakhstan, Astana, Kazakhstan; 5 Department of Public Health, Al-Farabi Kazakh National University (KazNU), Almaty, Kazakhstan; 6 Multidisciplinary City Hospital No. 1, Astana, Kazakhstan; 7 Department of Biomedical Sciences, School of Medicine, Nazarbayev University, Astana, Kazakhstan; 8 Interdisciplinary Sports Research, Center for Genetics and Life Sciences, Sirius University of Science and Technology, Sochi, Russia; 9 Faculty of Medicine and Health, Al-Farabi Kazakh National University (KazNU), Almaty, Kazakhstan; 10 Department of Normal Physiology, First Moscow State Medical University (Sechenov University), Moscow, Russia

**Keywords:** Kazakhstan, male-to-female ratio, prevention strategies, regional distribution analysis, suicide epidemiology, temporal trends

## Abstract

**Introduction:**

Despite some recent declines, suicides remain a major public health issue in Kazakhstan, with rates persistently above global benchmarks. This study provides the first integrated characterization of temporal trends, demographic characteristics, and regional distribution of suicide mortality in Kazakhstan.

**Materials and methods:**

This epidemiological analysis examined suicide mortality data over a 13-year period (2012–2024). Data were provided by the Republican State Enterprise “Forensic Examination Center of the Ministry of Justice of the Republic of Kazakhstan” and the State Institution “Committee on Legal Statistics and Special Records of the Prosecutor General’s Office.

**Results:**

Between 2012 and 2024, 46,546 suicides were registered in Kazakhstan. Absolute suicides decreased by 15.8% despite 19.3% population growth. By 2023–2024, SR = 17.8 (ASSR = 18.2) – more than 65% above the WHO global average. The male-to-female ratio was 3.8:1 (ASSRs: men 29.9, women 7.4). Among men, peaks occurred at 25–29 (SR = 38.6) and 45–54 (SR = 49.9); among women, the first peak was at 18–24 (SR = 10.6). Regional SRs ranged from 11.0 to 29.2 per 100,000. The aging index was the strongest demographic ecological correlate of regional SR (*r* = 0.84, *p* ≤ 0.0001). Annual sunshine duration showed a significant inverse association with SR (*β* = −0.814, 95% CI [−1.221; −0.407], *p* = 0.001) in the regional regression model, while average salary was not significant.

**Conclusion:**

Regional demographic restructuring – particularly population aging concentrated in northern regions – is the primary structural correlate of regional suicide variability. Annual sunshine duration emerged as a significant ecological correlate in the regression model specifically testing environmental and economic factors. These findings provide an evidence base for targeted, region- and population-specific prevention measures.

## Introduction

Suicide is one of the leading preventable causes of death worldwide, with more than 700,000 deaths occurring annually, according to the World Health Organization (WHO), accounting for roughly 1 in every 100 deaths (1). Because suicides disproportionately affect younger age groups, the associated loss in years of life lived (YLL) and disability-adjusted life years (DALYs) is substantially greater than that implied by mortality counts alone, making suicide prevention a priority for public health systems worldwide (1). Suicide rates per 100,000 population (SR) vary significantly worldwide, generally ranging from below 5 to over 40 deaths (1). Understanding this epidemiological heterogeneity and identifying population-specific risk factors are important steps for the targeted implementation of preventive measures (1, 2).

Kazakhstan is the largest country and a leading economy in Central Asia, with a population exceeding 20 million as of 2024. Following the collapse of the Soviet Union in 1991, the country underwent profound demographic and economic transformation while persistently recording some of the highest suicide rates globally (3–5). According to the Global Burden of Disease Study 2021, Kazakhstan ranks among the top 20 countries worldwide by age-standardized suicide rate, with rates consistently exceeding 150% of the WHO global average (6). The post-independence period of the 1990s saw a dramatic escalation in suicide mortality, with rates peaking at over 30 per 100,000 population, driven by the socioeconomic collapse that followed dissolution of the Soviet Union (3–5). While subsequent decades brought a gradual decline, rates have remained persistently elevated compared to global benchmarks. Alcohol consumption is a well-documented co-factor in Kazakhstani suicides – population-wide data indicate that approximately 20% of completed suicides involve acute alcohol use (7), a pattern that interacts with cultural norms, gender roles, and the limited availability of mental health infrastructure in rural and northern regions.

Although a number of studies have documented a consistently high suicide rate in Kazakhstan compared to global figures (1, 6–9), prior studies have been limited in scope: most focused on single years or short time windows, specific demographic subgroups, or individual regions, and none have simultaneously integrated temporal trends, age- and sex-stratified mortality, and regional geospatial patterning within a single analytical framework. Critically, no previous study has examined the relationship between demographic predictors – such as population aging and regional sex ratios – and suicide rates across Kazakhstan’s administratively distinct regions. Furthermore, the data underlying this analysis – drawn directly from the Republican Forensic Examination Center and the Committee on Legal Statistics of the Prosecutor General’s Office – represent the most complete official record of completed suicides available in Kazakhstan and have not previously been used for a systematic epidemiological characterization of this scale. Addressing this gap is of direct policy relevance: without a comprehensive, demographically sensitive baseline, it is not possible to identify the most vulnerable populations or regions, formulate evidence-based hypotheses about the driving factors, or prioritize targeted prevention efforts within Kazakhstan’s National Public Health Program.

A north–south gradient in suicide rates within individual countries is a widely observed phenomenon across diverse climate zones. In temperate and subarctic settings, this pattern has been linked to shorter daylight hours, higher rates of alcohol consumption, lower population density, and more limited access to mental health care in northern regions (10–19). Comparable intra-country gradients have been documented in Sweden, Finland, France, Russia, the United States, and Thailand (10–19), while in South Korea, peripheral rural regions – rather than strictly northern ones – bear the highest burden, reflecting a rural–urban rather than a purely latitudinal divide (20–23). These cross-national patterns provide an important comparative frame for interpreting Kazakhstan’s regional heterogeneity.

This study presents an epidemiological analysis of completed suicides in Kazakhstan over a 13-year period (2012–2024). We examined temporal trends, demographic characteristics, and regional distribution of suicide mortality. This analysis provides an in-depth characterization of the suicide burden in Kazakhstan, highlighting potential areas for targeted prevention efforts. The findings are intended to serve as an evidence base for the development and prioritization of region- and population-specific suicide prevention measures within Kazakhstan’s national public health strategy.

## Methods

### Data

Aggregated anonymized data on suicide counts for the period from January 1, 2012, to December 31, 2024, were obtained from the Research Institute of the branch of the Republican State Enterprise “Ministry of Justice of the Republic of Kazakhstan” and the State Committee “Center for Judicial Statistics and Special Accounting of the Prosecutor General’s Office of the Republic of Kazakhstan,” which conduct judicial proceedings of completed suicides in Kazakhstan. The Republican State Enterprise on the Right of Economic Management “Kazhydromet” of the Ministry of Ecology and Natural Resources of the Republic of Kazakhstan for providing data on annual sunshine duration (number of hours of solar insolation per year) across the country.

All authors confirm that all methods were carried out in accordance with relevant guidelines and regulations, including the Declaration of Helsinki, the International Ethical Guidelines for Biomedical Research Involving Human Subjects (CIOMS), and the Good Clinical Practice (GCP) guidelines developed by the International Council for Harmonisation (ICH GCP E6 R3).

Ethical approval was granted by the Local Ethics Committee of the Department of Science and Innovation at Al-Farabi Kazakh National University (IRB – А1685), under the study title “Gut Microbiome as a Biomarker of Suicidal Risk: A Comprehensive Analysis and Preventive Strategies in Public Health.” Date of approval: July 17, 2025. The present analysis of aggregated, anonymized suicide mortality data from official state registries was conducted under the same research program covered by IRB – А1685, and did not involve the collection of any biological material or individual-level personal data.

Since this study was based exclusively on aggregated, anonymized mortality statistics obtained from official state registries of the Republic of Kazakhstan, individual patient data were not accessed at any stage of the research. Accordingly, informed consent was not required.

The obtained data were stratified by: (1) calendar year (2012–2024); (2) geographic region/city; (3) age groups (5–14, 15–17, 18–24, 25–29, 30–34, 35–44, 45–54, 55–64, 65–74, 75 + years); (4) gender (male, female); and (5) method of suicide. Geographic regions were defined by 17 administrative-territorial units and 3 major cities (Astana, Almaty, Shymkent).

### Statistical analysis

Suicide rates per 100,000 population (SR) and age-specific rates were calculated using annual population estimates provided by the Committee on Legal Statistics and Special Accounts of Kazakhstan. Rates were calculated using the population aged 5 years and older as the denominator, consistent with the lower age bound of the outcome data (ICD-10 ×60-X84, ages 5–75+). Age-standardized suicide rates (ASSRs) were calculated using the WHO World Standard Population 2000–2025 as a reference.

Time trends were analyzed using linear regression. Confidence intervals (95% CI) for mean statistics were calculated using the bootstrap resampling method with replacement (999 iterations). The Spearman correlation coefficient (*r*) was calculated to assess relationships between SRs and explanatory factors. Associations were considered significant at *p* < 0.05. Given the exploratory nature of the correlation analyses in Table 1, a Benjamini-Hochberg false discovery rate (FDR) correction was applied; adjusted *p*-values are reported alongside the original values. Statistical analysis was performed in Python v3.12.2 using the SciPy v1.15.1 and Matplotlib v3.8.2 libraries.

**Table 1:** Regional correlates of suicide mortality.

Variable 1	Variable 2	Spearman’s r	*p*-value
**Avg. pop. Age: Pop M/R**	**Suicides per 100,000**	***r* = 0.89**	***p* ≤ 0.0001**
Avg. pop. Age: Pop M/R	Suicides per 100,000, age 25–29	*r* = 0.72	*p* ≤ 0.001
Avg. pop. Age: Pop M/R	Suicides per 100,000, age 30–34	*r* = 0.67	*p* ≤ 0.001
**Avg. pop. Age: Pop M/R**	**Suicides per 100,000, age 35–44**	***r* = 0.9**	***p* ≤ 0.0001**
**Avg. pop. Age: Pop M/R**	**Suicides per 100,000, age 45–54**	***r* = 0.75**	***p* ≤ 0.0001**
Avg. pop. Age: Pop M/R	Suicides per 100,000, age 55–64	*r* = 0.74	*p* ≤ 0.001
Avg. pop. Age: Pop M/R	Suicides per 100,000, age 65–74	*r* = 0.63	*p* ≤ 0.01
Avg. pop. Age: Pop M/R	Suicides per 100,000, age 75+	*r* = 0.6	*p* ≤ 0.01
**Avg. pop. Age**	**Suicides per 100,000**	***r* = 0.84**	***p* ≤ 0.0001**
Avg. pop. Age	Suicides per 100,000, age 25–29	*r* = 0.69	*p* ≤ 0.001
Avg. pop. Age	Suicides per 100,000, age 30–34	*r* = 0.61	*p* ≤ 0.01
**Avg. pop. Age**	**Suicides per 100,000, age 35–44**	***r* = 0.8**	***p* ≤ 0.0001**
Avg. pop. Age	Suicides per 100,000, age 45–54	*r* = 0.6	*p* ≤ 0.01
Avg. pop. Age	Suicides per 100,000, age 75+	*r* = 0.66	*p* ≤ 0.01
Pop. thousands	Total number of suicides	*r* = 0.65	*p* ≤ 0.01
Pop. thousands	Suicides per 100,000, age 75+	*r* = −0.63	*p* ≤ 0.01
% of urban population	Hanging, %	*r* = −0.62	*p* ≤ 0.01
Income (Tg)	Hanging, %	*r* = −0.65	*p* ≤ 0.01
Death rate	Suicides per 100,000	*r* = 0.73	*p* ≤ 0.001
**Death rate**	**Suicides per 100,000, age 35–44**	***r* = 0.79**	***p* ≤ 0.0001**
Death rate	Suicides per 100,000, age 45–54	*r* = 0.59	*p* ≤ 0.01
Death rate	Suicides per 100,000, age 55–64	*r* = 0.63	*p* ≤ 0.01
Death rate	Suicides per 100,000, age 75+	*r* = 0.61	*p* ≤ 0.01
Birth rate	Suicides Per 100 thousands	*r* = −0.69	*p* ≤ 0.001
Birth rate	Suicides per 100,000, age 25–29	*r* = −0.6	*p* ≤ 0.01
Birth rate	Suicides per 100,000, age 35–44	*r* = −0.6	*p* ≤ 0.01
Birth rate	Suicides per 100,000, age 75+	*r* = −0.57	*p* ≤ 0.01
**Aging index**	**Suicides per 100,000**	***r* = 0.84**	***p* ≤ 0.0001**
Aging index	Suicides per 100,000, age 25–29	*r* = 0.67	*p* ≤ 0.001
Aging index	Suicides per 100,000, age 30–34	*r* = 0.6	*p* ≤ 0.01
**Aging index**	**Suicides per 100,000, age 35–44**	***r* = 0.82**	***p* ≤ 0.0001**
Aging index	Suicides per 100,000, age 45–54	*r* = 0.62	*p* ≤ 0.01
Aging index	Suicides per 100,000, age 75+	*r* = 0.63	*p* ≤ 0.01
Migration in/out	Hanging, %	*r* = −0.66	*p* ≤ 0.01

“Avg. pop. age:Pop M/R” represents the interaction term between the region’s average population age and the male-to-female ratio in the region. Bold values indicate the strongest statistically significant correlations (|r| ≥ 0.75, p < 0.001).

The multivariable linear regression model included average salary and annual sunshine duration as the two explanatory variables. This two-variable model was constructed with the specific and limited aim of comparing the relative contribution of an environmental factor (insolation) versus a socioeconomic factor (salary), as these represent conceptually distinct and potentially policy-relevant domains. We acknowledge that this modeling choice has an important limitation: the observed association between sunshine duration and regional suicide rates may not be independent of the regional demographic structure. Future studies should test this in a comprehensive multivariate model incorporating average population age, aging index, and regional sex ratio alongside environmental and economic variables.

All associations reported in this study are correlational in nature and should not be interpreted as causal or predictive relationships, as the study design is ecological and cross-sectional.

Risk categories were defined empirically based on the observed distribution of regional rates in the 2023–2024 data: the national average SR was 17.8, and thresholds were set at approximately ±25% and ±55% of the national average to create four risk strata: low (<17), medium (17–22), high (>22), and critical (>27) per 100,000. These thresholds are not derived from externally validated epidemiological benchmarks and the classification should be interpreted as a descriptive heuristic rather than a formally validated risk stratification tool.

### Strength and limitations

This is the first study to provide a thorough, simultaneous characterization of temporal trends, demographic characteristics, and regional distribution of suicide mortality in Kazakhstan. However, this study has several limitations. First, this study uses pooled data reported from different regions, and the rigor of forensic examinations may vary between areas, potentially leading to inconsistent accuracy in suicide case estimates across regions. Second, individuals who died outside their permanent place of residence may have been registered in the region of death rather than their region of residence, which could have impacted regional estimates given Kazakhstan’s high internal migration flows. Third, the current analysis lacks data on comorbidities, particularly psychiatric conditions, greatly constraining the ability to interpret the revealed patterns. Fourth, the ecological design of the regional analyses is susceptible to residual confounding by variables not included in the models – including ethnic composition, religion, alcohol consumption rates at the regional level, access to psychiatric services, and cultural attitudes toward help-seeking. Fifth, this study did not conduct formal spatial autocorrelation analysis (e.g., Moran’s I or LISA statistics); the apparent north–south clustering visible in Figure 1A suggests that spatial autocorrelation may be present, which would violate the independence assumption underlying the Spearman correlations and regression analyses presented. Sixth, regional age-specific rates in Table 2 are based on a two-year reference period (2023–2024) and may be statistically unstable for small regions and narrow age strata with few observed events – in particular, estimates for Ulytau region (e.g., 196.4 per 100,000 among men aged 75+) and cells with zero events should be interpreted with caution. Seventh, these findings illustrate that population-level demographic and environmental correlates identified in the present study may coexist with – and potentially be amplified by – individual clinical and psychiatric vulnerabilities that cannot be captured in aggregated mortality datasets.

**Figure 1:**
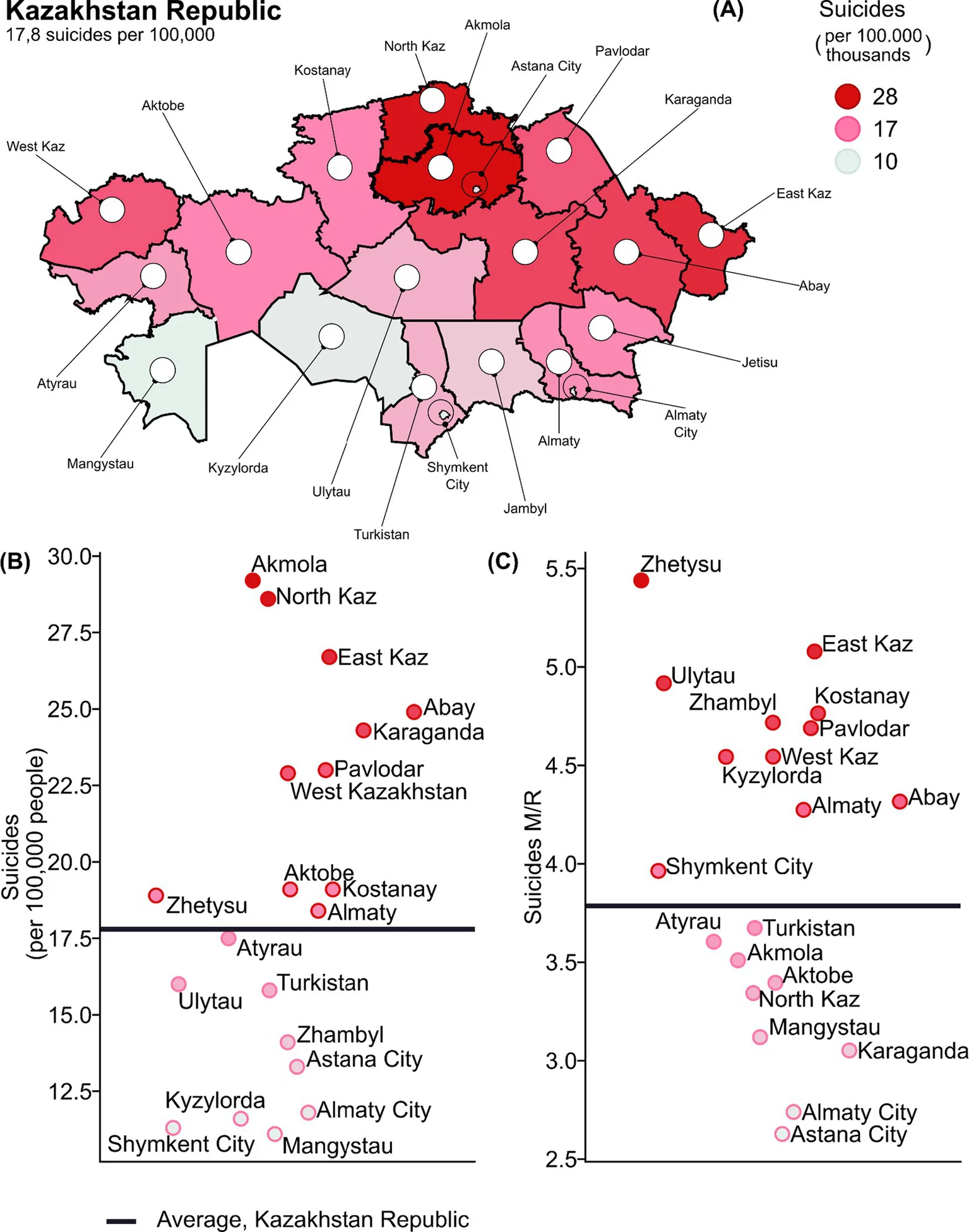
Regional variation in suicide mortality rates and male-to-female (M/F) ratios of suicides in Kazakhstan. **(A)** Geospatial distribution of suicide rates per 100,000 population; **(B)** Distribution of suicide rates per 100,000 population; **(C)** Distribution of male-to-female ratios.

**Table 2:** Age-specific and age-standardized suicide rates per 100,000 population in Kazakhstan (2023–2024).

Region/City	5–14	15–17	18–24	25–29	30–34	35–44	45–54	55–64	65–74	75+	ASSR
Age-specific and age-standardized suicide rates per 100,000 (MEN)											
Kazakhstan Republic	2.1	16.2	31.5	38.6	40	46.8	49.9	44.4	40.1	51.3	29.8
Abay region	3.7	13.1	43.3	42.1	57	65.7	66.3	65	52	55.8	39.4
Akmola region	0	22	43.1	68.8	55.6	76.4	68	68.8	55.5	63.4	43.9
Aktobe region	2.1	8.7	46.1	46.8	49.3	51.1	46.7	33.6	47.9	59.2	31.8
Almaty region	3.1	21	28.8	33.5	42	47.8	50.5	54.7	39.5	49.1	30.9
Atyrau region	2.5	21.8	39.7	52.1	43.5	40	47.9	35.3	62.5	0	30.1
West Kaz region	1.6	25.3	44.9	42.9	44	63.4	68	57.4	37.1	95.6	38.9
Zhambyl region	3	19.3	30.8	26.9	28.4	38.5	48.5	34.8	35.9	29.6	25.4
Zhetysu region	2.8	21.3	21.6	45.7	41.9	50.5	56.4	54.2	42	93.8	33.8
Karaganda region	1.1	11.9	38.7	49.4	56.3	60.9	59.4	54.6	46.3	53.8	36.3
Kostanay region	0	23.7	21	60.4	37.8	51.2	43.2	41.2	48.4	36.7	30.4
Kyzylorda region	2.1	8.2	19.3	14.4	25.9	33.6	40.1	34.5	24.3	55.4	20.8
Mangystau region	2.1	18.7	14.4	23.4	33.3	26.6	33.6	34.2	23.6	0	19.1
Pavlodar region	0	0	17.9	47.4	64.9	64.6	78.2	47.3	37	66.6	35.8
North Kaz region	0	18	29.7	55.8	51.6	72.6	76.1	57.5	55.6	86	41.2
Turkistan region	3.7	22.4	29.2	30.9	35.9	40.1	55.6	42.5	37	32.9	28.4
Ulytau region	9.3	0	23.6	42.5	23.7	52.7	31.5	37.2	36.6	196.4	30.5
East Kaz region	1.9	6.8	50.7	54.9	73.8	88.2	55.6	51.1	50	74.6	42.6
Astana city	3	6.8	30.9	37.9	27.3	25.7	27.5	32.1	25.4	39.5	20.6
Almaty city	0.5	12.6	23.4	21	30.6	26.1	23.9	29.1	32	28.8	18.3
Shymkent city	1.6	21.2	23.5	33.9	21.9	31.3	43.1	28.4	15.3	32.6	21.7
Age-specific and age-standardized suicide rates per 100,000 (WOMEN)											
Kazakhstan Republic	1.2	8.7	10.6	9.9	10.6	10.3	10.3	7.7	8.2	**12**	7.4
Abay region	2	0	20.5	6.7	10.6	9.9	14.6	10.2	21.3	12.4	8.7
Akmola region	0	0	19.9	14.4	22.7	14.1	17.8	22	10.8	9.6	11.1
Aktobe region	0	4.6	11	24.6	11.4	10.6	14.7	11.9	6.2	13.2	9
Almaty region	0.7	14.7	12	14.9	11.2	12.7	4.6	5.3	8.2	4.6	7.7
Atyrau region	1.3	11.4	10.6	9.1	14.8	10.4	5.5	5.9	10.2	24.2	7.7
West Kaz region	3.4	0	7.9	10.3	7.9	14.2	9.4	11.7	6.9	12.9	7.4
Zhambyl region	0	2.9	9.8	11.8	10.1	7.8	8.7	6.8	5.1	0	5.6
Zhetysu region	3	0	7.7	11.3	4.5	13	4.7	7.8	7.6	0	5.8
Karaganda region	1.1	4.2	13.7	9.3	22.4	19	24.2	7.1	10.4	11.7	11
Kostanay region	0	6.2	9.7	4.5	14	9.7	7.2	3.5	4.6	21.8	6.1
Kyzylorda region	0	8.7	0	4.1	7.2	7.7	6.5	5.1	4.3	0	4.1
Mangystau region	1.1	9.9	9.2	7.9	6.7	3.8	7.8	9.6	10.8	0	5.7
Pavlodar region	1.7	12.8	7.4	0	7.8	10.6	13.5	7.8	5.3	17.8	7
North Kaz region	2.8	0	0	16.1	17.5	17.8	22.3	15.6	12.4	21.9	10.7
Turkistan region	1.6	13.3	14.3	10	18.7	9.1	5.8	7	4	9.4	7.8
Ulytau region	0	0	0	0	0	0	14.5	7.8	0	42.2	3.6
East Kaz region	0	14.2	4.7	16.9	8.2	14.7	10.2	5.9	8.7	14.9	8.1
Astana city	1.6	10.4	17.4	11.7	8.2	4.8	7.5	7.8	5.1	23.1	7.3
Almaty city	1.1	4.4	10.5	7.1	5.9	9.7	7.4	3.5	7	8.2	5.5
Shymkent city	0.8	19.1	6.5	2.3	4.2	4.9	6.7	4.1	13.5	0	5.3

## Results

### Temporal trends in suicide rates (2012–2024)

Between 2012 and 2024, 46,546 completed suicides were registered in Kazakhstan, averaging 3,580 cases per year (95% CI: 3,390-3,760). The data are presented in Table 3 and Figure 2.

**Table 3:** Temporal trends in completed suicides in Kazakhstan (2012–2024).

Year	Population of Kazakhstan Republic	Number of suicides (Ages 5–75+)	SR	ASSR	Absolute change from previous year	% Change from previous year	Period classification
2012	16,791,721	4,049	20.6	-	-	-	Phase 1: Observation start
2013	17,035,010	4,175	21.2	-	+126	+3.1%	Decline
2014	17,287,554	3,860	19.6	-	−315	−7.6%	Decline
2015	17,541,804	3,924	19.9	-	+64	+1.7%	Decline
2016	17,792,672	3,609	18.3	-	−315	−8.0%	Decline
2017	18,036,224	3,423	17.4	-	−186	−5.2%	Decline
2018	18,275,234	3,308	16.8	-	−115	−3.4%	Stabilization
2019	18,512,781	3,426	17.4	-	+118	+3.6%	Stabilization
2020	18,754,943	2,928	14.9	-	−498	−14.5%	COVID-19
2021	19,000,687	3,511	17.9	-	+583	+19.9%	Post-pandemic
2022	19,634,983	3,231	16.5	-	−280	−8.0%	Normalization
2023	19,766,807	3,694	18.7	19.2	+463	+14.3%	Stabilization
2024	20,033,842	3,408	17.0	17.3	−286	−8.4%	Stabilization
	Overall trend: +3,366,900	Total 2012–2024: 46,546	Avg. 2023–2024: 17.8	Avg. 2023–2024: 18.2	Overall trend: −641	Overall trend: −15.8%	

**Figure 2:**
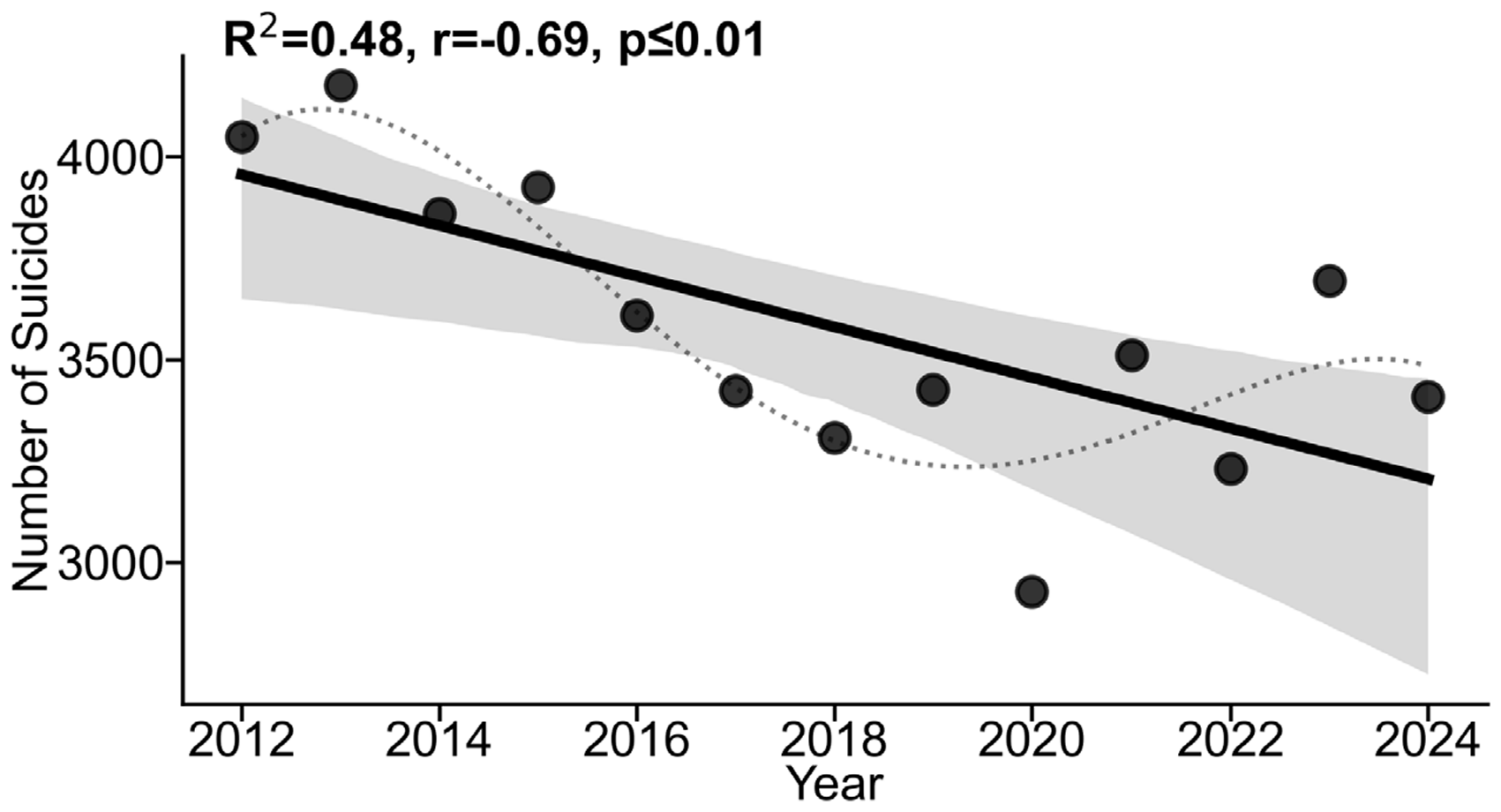
Temporal trends in number of completed suicides in Kazakhstan (2012–2024).

Based on an analysis of time trends, several phases of suicide mortality can be identified: (1) an initial decline was observed between 2012 and 2017; (2) followed by relative stabilization in 2017–2019; (3) in 2020, during the COVID-19 pandemic, an unexpected 14.5% decrease in cases was recorded (from 3,426 to 2,928), which is notable in that it runs counter to the increases or stable rates reported in several high-income countries during the COVID-19 pandemic (24, 25), however, a formal statistical cross-national comparison was not conducted in this study, and this observation should be interpreted with caution; (4) in 2021, a post-pandemic relative increase of 19.9% (from 2,928 to 3,511 cases) was observed; and (5) finally, in 2022–2024, the figures returned in line with the trend established in 2017–2019.

Overall, over the study period (2012–2024), a reduction of 15.8% (absolute decrease of 641 cases) in the absolute number of suicides was observed. Notably, this decline occurred against a backdrop of stable and significant population growth of 19.3% (absolute increase of >3 million people). By the end of the study period, the suicide rate (SR) reached 17.8 per 100,000 population (95% CI: 15.4–19.9 across regions), and the age-standardized suicide rate (ASSR) was 18.2 (95% CI: 17.1–21.2 across regions).

### The age distribution of suicides by sex

Among the 46,546 completed suicides registered between 2012 and 2024, 37,203 (79.9%) were male and 9,343 (20.1%) were female. The male-to-female ratio at the end of the study period was 3.8:1 (95% CI: 3.5–4.6). Age distribution showed significant differences in rates between males and females. The data are presented in Table 4 and Figure 3.

**Table 4:** Average age distribution of suicides by sex in Kazakhstan (2023–2024).

Age group (years)	Avg. population men, %	Avg. population women, %	Avg. suicides men, *n* (%)	Avg. suicides women, *n* (%)	Age Abs. M/F ratio	Men suicides per 100,000	Women suicides per 100,000
5–14	22.4	20	42 (1.5%)	23 (3.1%)	1.8	2.1	1.2
15–17	5.8	5.2	82 (2.9%)	42 (5.6%)	2	16.2	8.7
18–24	9.9	9	273 (9.7%)	88 (11.8%)	3.1	31.5	**10.6**
25–29	7.2	6.5	242 (8.6%)	60 (8.1%)	4	**38.6**	9.9
30–34	8.6	8	302 (10.8%)	78 (10.6%)	3.9	40.0	10.6
35–44	16.2	15.3	661 (23.5%)	146 (19.6%)	4.5	46.8	10.3
45–54	12.3	12.6	535 (19%)	120 (16.1%)	4.5	**49.9**	10.3
55–64	10	11.4	389 (13.9%)	81 (10.9%)	**4.8**	44.4	7.7
65–74	5.5	7.9	194 (6.9%)	60 (8.1%)	3.2	40.1	8.2
75+	2	4.1	88 (3.1%)	46 (6.1%)	1.9	**51.3**	**12.0**
					Kazakhstan = 3.8	ASSR = 29.9	ASSR = 7.4

Bold values indicate the highest or particularly elevated age-specific values within the corresponding indicators.

**Figure 3:**
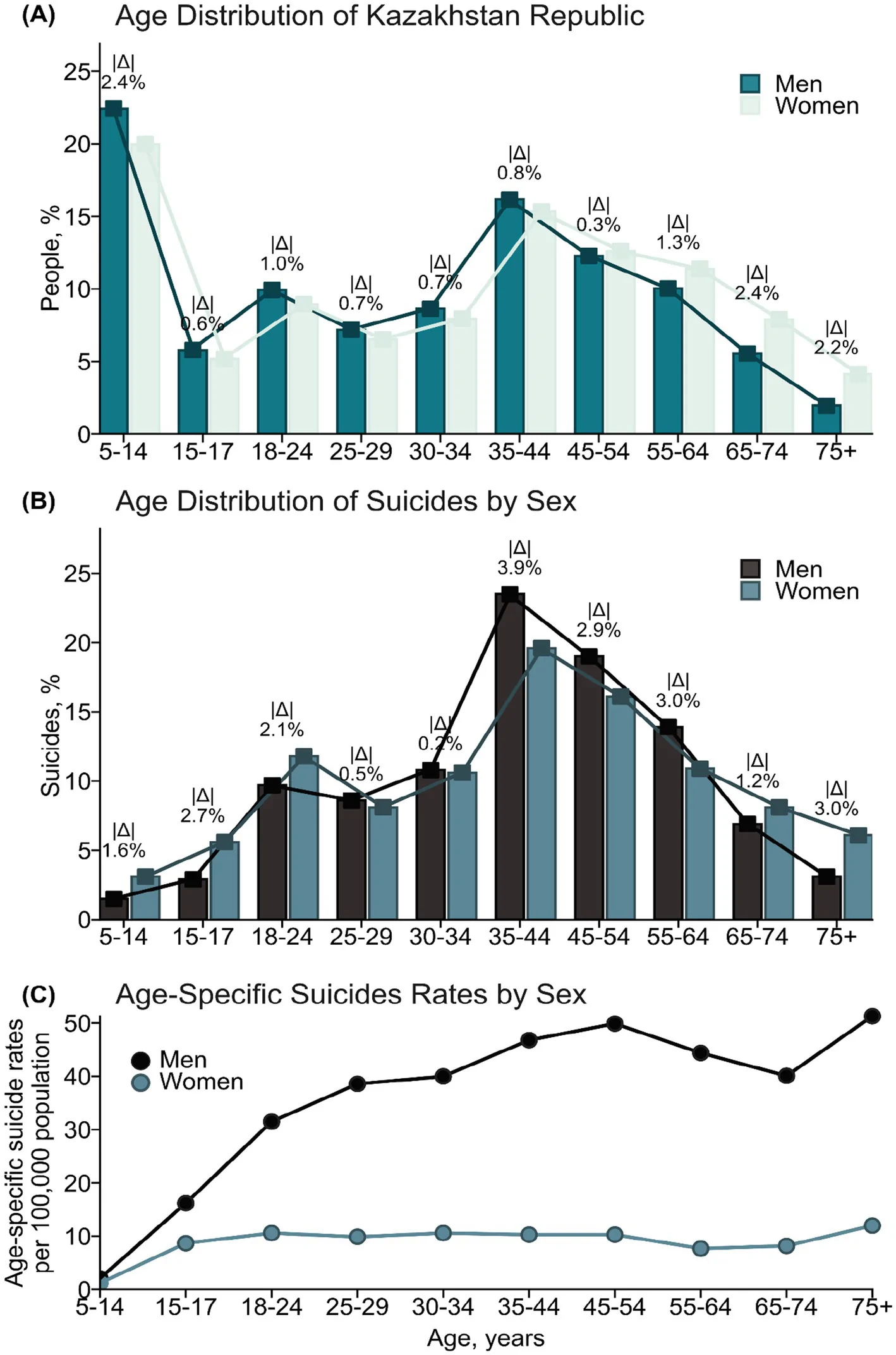
Age distribution of the Kazakhstan population and average age distribution of suicides by sex in Kazakhstan (2023–2024). **(A)** Bar plot of age distribution of the Kazakhstan population (%). **(B)** Bar plot of age distribution of suicides by sex (%). **(C)** Line plot of age-specific suicide rates per 100,000 population for men and women.

Across all age groups, there was an absolute predominance of deaths among men compared to women (ASSR = 29.9 vs. 7.4). However, the relative distribution of suicides across age groups differed by gender. In the 5–17 age group, the proportion of suicides among women was higher: 8.7% of all female cases occurred in this age group, compared to 4.4% among men. A similar trend was observed in the 75 + age group, which accounted for 6.1% of all female deaths and 3.1% among men. For the older age group, this observation may be explained by the longer life expectancy of women in Kazakhstan (life expectancy: men – 71.33 years, women – 79.42 years (26)).

The highest number of suicides among individuals over 35 years of age occurred in the 35–44 age group: 23.7% (95% CI: 22.4–25.3) among men and 20.3% (95% CI: 15.8–21.8) among women, with a male-to-female ratio of 4.5:1. This is generally consistent with the demographic distribution of the working-age population, where the majority of individuals are also aged 35–44.

Analysis of age-specific mortality rates revealed that the highest suicide risk for both men and women occurred in the oldest age group (75 + years), with SRs reaching 51.3 among men and 12.0 among women. However, the age-specific patterns leading to this peak differed markedly by gender. Men exhibited a more complex age-related risk trajectory, characterized by two distinct peaks before the age of 75. The first occurred in early adulthood (25–29 years), with a SR of 38.6, followed by a second, more pronounced peak in middle age (45–54 years), where the SR rose to 49.9. In contrast, women demonstrated a relatively even distribution of risk across the lifespan, though with an earlier initial increase. A single prominent peak was observed in young adulthood (18–24 years), with a rate of 10.6, after which rates remained comparatively stable before rising again in old age.

### Regional differences in suicide rates and male-to-female (M/F) ratios in Kazakhstan

Suicide mortality rates varied drastically across regions, ranging from 28.0 per 100,000 population in north regions of Kazakhstan to 11.0 in south regions and the major cities. The data are presented in Table 5 and Figure 1.

**Table 5:** Regional distribution of suicide mortality in Kazakhstan (2023–2024).

Region/City	Avg. population (thousands)*	Avg. annual suicides	Avg. rate per 100,000	Avg. hanging %	Avg. Sober %	M/R	Risk group
Kazakhstan Republic	19900.3	3,551	17.8	78.2	80.4	3.8	Medium
Abay region	608.9	151.5	24.9	84.4	84.4	4.3	High
Akmola region	788.0	230.0	29.2	83.2	72.4	3.5	**Critical**
Aktobe region	933.8	178.0	19.1	73.8	78.7	3.4	Medium
Almaty region	1518.5	279.5	18.4	85.1	84.4	4.3	Medium
Atyrau region	698.6	122.0	17.5	71.0	86.0	3.6	Medium
West Kaz region	690.7	158.0	22.9	86.7	71.0	4.5	High
Zhambyl region	1220.4	171.5	14.1	85.5	82.7	4.7	Low
Zhetysu region	698.4	132.0	18.9	87.3	78.9	5.4	Medium
Karaganda region	1135.1	275.5	24.3	71.5	74.4	3.1	High
Kostanay region	831.1	158.5	19.1	87.5	89.2	4.8	Medium
Kyzylorda region	837.8	97.0	11.6	86.6	87.6	4.5	Low
Mangystau region	777.0	86.5	11.1	72.0	79.3	3.1	Low
Pavlodar region	754.4	173.5	23.0	79.0	73.5	4.7	High
North Kaz region	532.1	152.0	28.6	83.8	79.3	3.3	**Critical**
Turkistan region	2130.7	336.5	15.8	83.3	90.1	3.7	Low
Ulytau region	221.5	35.5	16.0	82.7	86.5	4.9	Low
East Kaz region	728.6	194.5	26.7	81.6	68.1	5.1	High
Astana city	1392.3	185.0	13.3	56.0	79.2	2.6	Low
Almaty city	2195.3	258.0	11.8	62.4	82.2	2.7	Low
Shymkent city	1207.1	136.5	11.3	75.2	88.9	4	Low

*Avg. parameters from 2023–2024; population statistic from stat.gov.kz.

The Akmola region and Northern Kazakhstan currently represent a critical risk zone (>27 per 100,000), approximately 1.5 times higher than the national average. Abay, West Kazakhstan, Pavlodar, East Kazakhstan, and Karaganda are classified as high-risk zones (>22 per 100,000). Medium-risk regions (17–22) include Aktobe, Almaty, Atyrau, Zhetysu, and Kostanay. Low-risk regions (<17) include Zhambyl, Kyzylorda, Mangystau, Turkestan, Ulytau, and the cities of Astana, Almaty, and Shymkent. These thresholds were defined empirically based on the distribution of regional rates around the national average (SR = 17.8) and should be interpreted as descriptive heuristics rather than formally validated risk strata.

Overall, approximately 6.5% of Kazakhstan’s population currently resides in the critical risk zone, 19.3% in the high-risk zone, 23.3% in the medium-risk zone, and 50.9% in the low-risk zone.

#### Regional variation in suicides male-to-female (M/F) ratios

The male-to-female ratio among suicide deaths showed significant regional heterogeneity (min-max range: 2.6–5.4). The highest gender disparity (M/*F* ≥ 4.8) was observed in the Zhetysu (5.4), East Kazakhstan (5.1), Ulytau (4.9), and Kostanay (4.8) regions. The lowest gender disparity (M/*F* < 3.5) was recorded in the cities of Astana (2.6) and Almaty (2.7), as well as in the Karaganda (3.1), Mangistau (3.1), North Kazakhstan (3.3), Aktobe (3.4), and Akmola (3.5) regions. The largest cities (Almaty and Astana) had the most balanced suicides gender ratios (<3). However, in Shymkent, however, this ratio was slightly higher than the national average (4.0 vs. 3.8).

#### Regional age-specific suicide mortality rates in Kazakhstan

Given the profound demographic heterogeneity of Kazakhstan’s population (27–30), we further analyzed regional age-specific suicide mortality rates. The data are presented in Table 2 and Figure 4.

**Figure 4:**
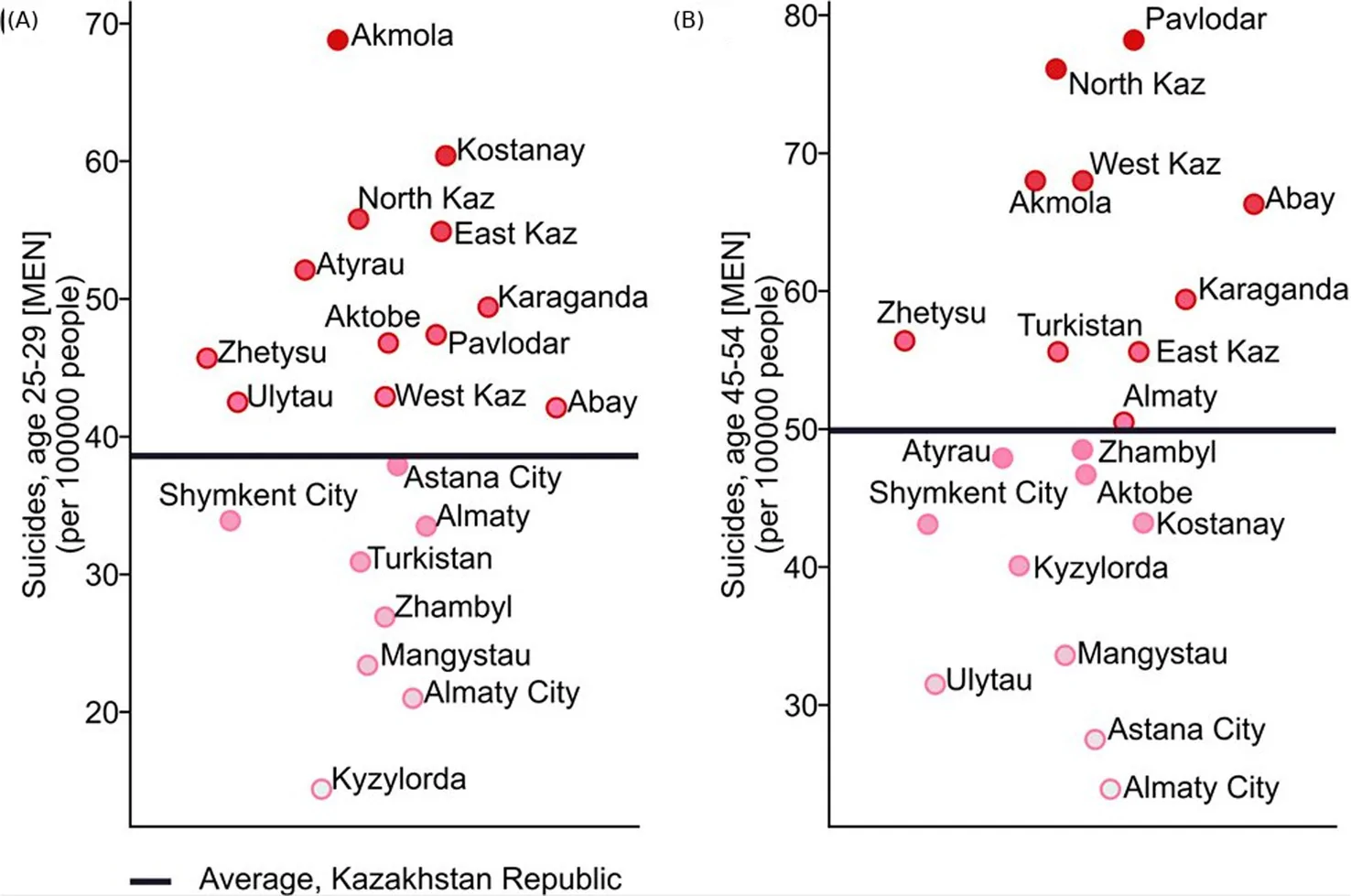
Regional age-specific suicide rates per 100,000 population in risk age groups. **(A)** Ages 25–29; **(B)** Ages 45–54.

It is worth noting that the lowest age-specific mortality in risk group of among men aged 45–54 years, in this study, was also observed in cities (<30 compared to the national average of 49.9), One plausible interpretation is that greater availability of psychiatric services and economic opportunities in urban centers may confer a protective effect – however, no direct measures of healthcare infrastructure, service utilization, or economic opportunity were collected in this study, and this interpretation remains speculative. It should be treated as a hypothesis to be tested in future research.

### Demographic and regional epidemiological suicide risk factors in Kazakhstan

The key ecological correlates of suicide mortality rates across Kazakhstan’s regions were the average age of the region population (*r* = 0.84; *p* ≤ 0.0001) and the population aging index (*r* = 0.84; *p* ≤ 0.0001). Upon further examination, the interaction between the average age of the region population and the proportion of men (“Avg. pop. age:Pop M/R”) provided an even stronger explanation of regional mortality rates (*r* = 0.89; *p* ≤ 0.0001). However, this composite term represents the product of two variables each independently associated with the outcome; the resulting correlation (*r* = 0.89) should be interpreted with caution, as this approach can inflate the correlation coefficient independently of any genuine interactive effect. This variable is presented as an exploratory descriptive finding rather than a formally tested statistical interaction. The data are presented in Table 1 and Figure 5.

**Figure 5:**
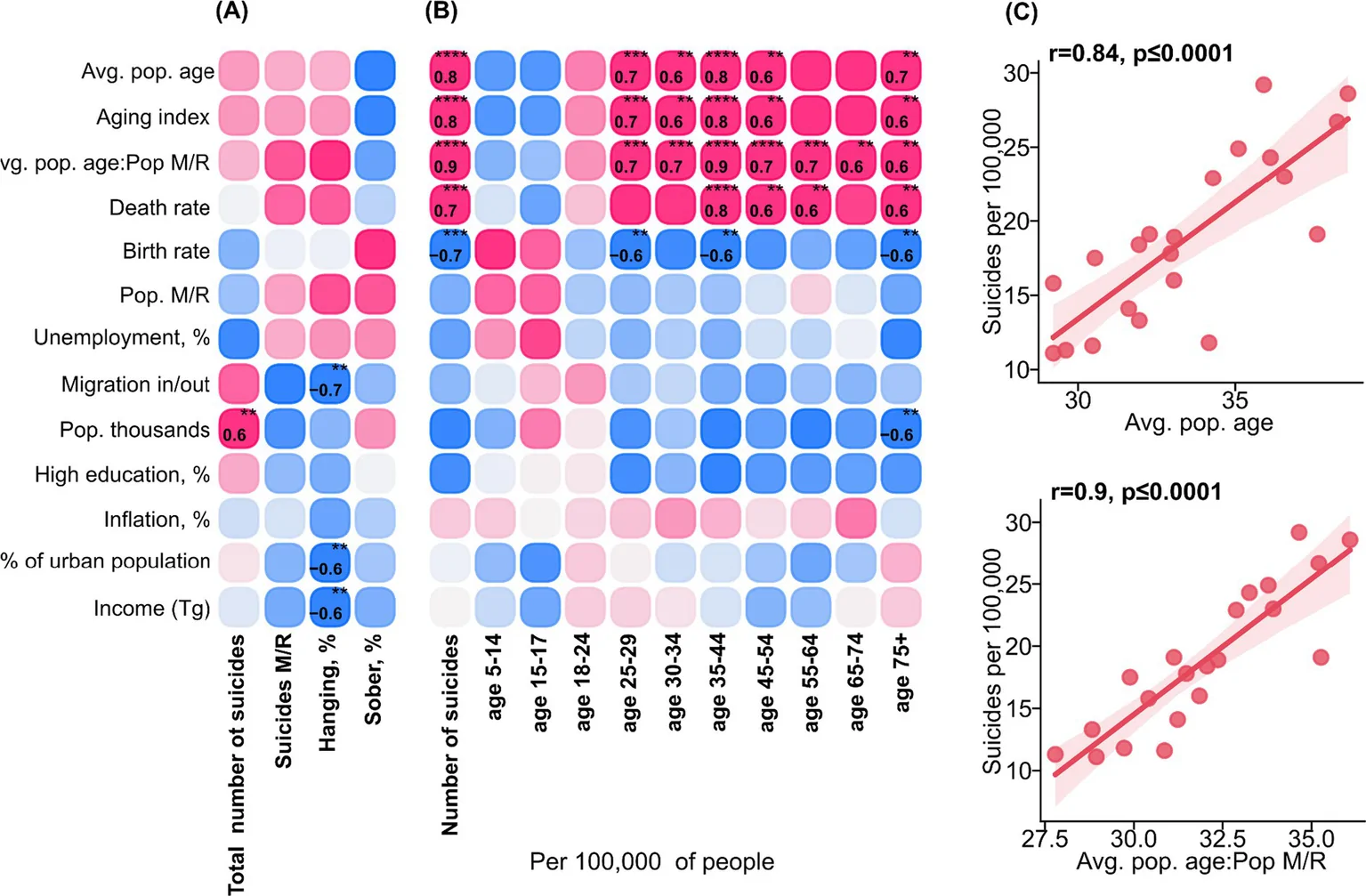
Demographic and regional epidemiological suicide risk factors in Kazakhstan. **(A)** Heatmap of correlations between suicide variables and explanatory factors; **(B)** Correlation between suicide rates per 100,000 population and explanatory factors; **(C)** Scatter plot with regression line showing the association between regional suicide rates and the best explanatory factors. Spearman’s r. **p ≤ 0.01; ***p ≤ 0.001; ****p ≤ 0.0001.

The regional sex ratio alone did not significantly correlate with the mortality rate (*p* > 0.05). Regional population size and the absolute number of suicides were positively correlated (*r* = 0.68; *p* < 0.001).

The correlation between overall death rate and suicide rate (*r* = 0.73, Table 1) should be interpreted with caution, as suicide deaths contribute to the overall death rate denominator; this part-whole relationship means that some degree of correlation is mathematically expected and does not necessarily reflect an independent epidemiological association. The overall death rate was calculated including suicide deaths, as separate data were not available in the registry.

The proportion of the region’s urban population was negatively correlated with the proportion of hanging as a suicide method (*r* = −0.62; *p* ≤ 0.01). Similarly, the average income level was negatively correlated with hanging (*r* = −0.65; *p* ≤ 0.01) and with the level of internal incoming migration to the region (*r* = −0.66; *p* ≤ 0.01).

In a multiple linear regression model with standardized variables, annual sun hours were inversely associated with suicide rate (*β* = −0.814, 95% CI [−1.221; −0.407], *p* = 0.001), whereas average salary was not significantly associated with suicide rate (*β* = −0.032, 95% CI [−0.439; 0.375], *p* = 0.864). Thus, of the two factors examined, insolation is a significant ecological correlate of regional suicide mortality observed across Kazakhstan; however, this association is ecological in nature and does not establish a causal or explanatory relationship. The possibility that this association is partially confounded by regional demographic structure – as northern regions are simultaneously older, more male-skewed, and less sunny – cannot be excluded. The data are presented in Table 6 and Figure 6.

**Table 6:** Multiple linear regression model: association of annual sun hours and average salary with regional suicide rates in Kazakhstan (standardized coefficients).

Variable	Adjusted coefficient, *β* [95% CI]	*p*-value
Salary	−0.032 [−0.439; 0.375]	0.864
Sun hours	−0.8143 [−1.221; −0.407]	0.001

**Figure 6:**
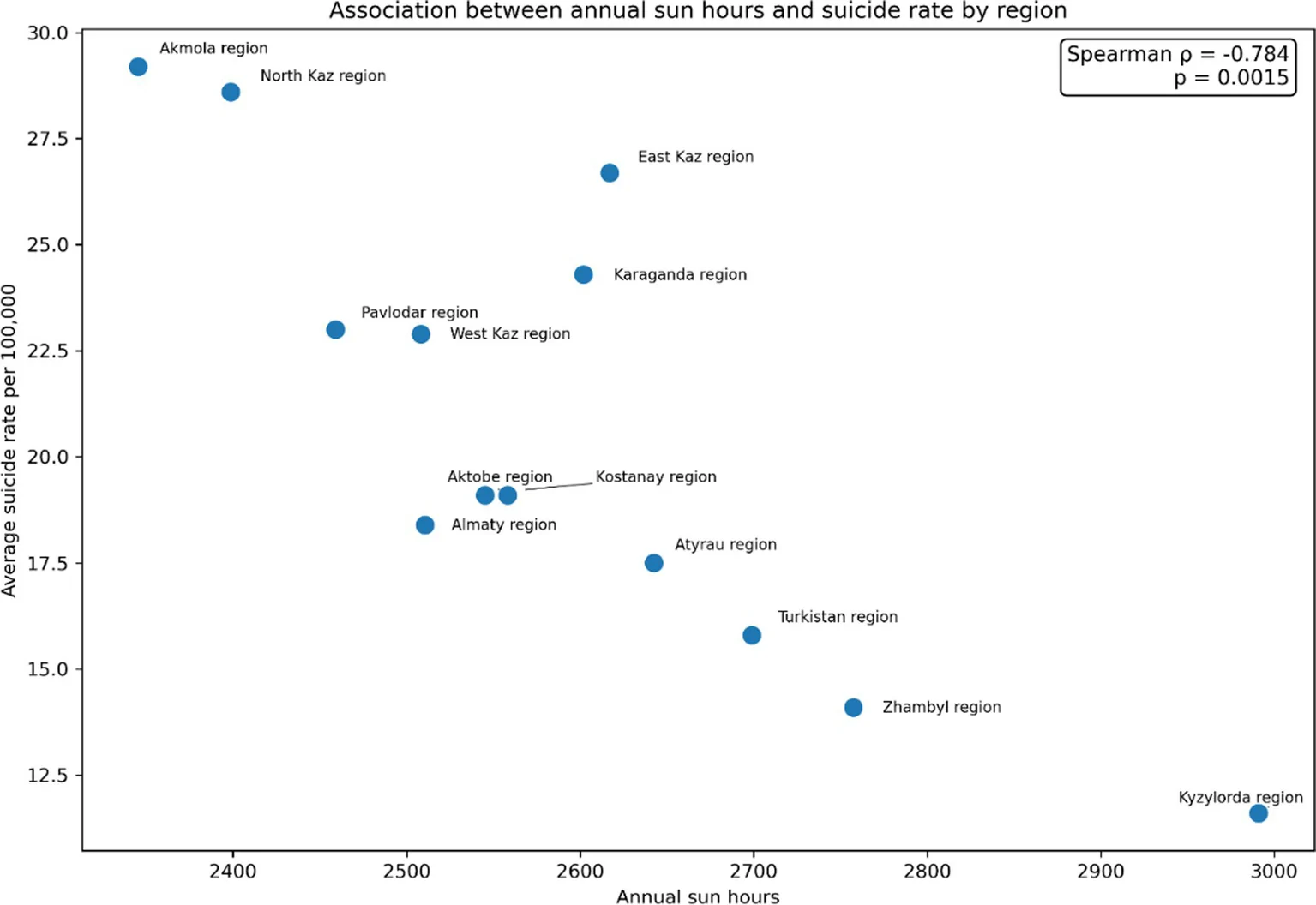
Association between annual sun hours and suicide rate by region. Spearman’s correlation coefficient was ρ = −0.784 at *p* = 0.0015, which shows a characteristic negative relationship – the fewer hours of sunshine in countries, the higher the suicide rate.

The Figure 6 shows the relationship between the annual number of hours of sunshine and the average suicide rate (per 100,000 population) across Kazakhstan regions.

The most disadvantaged regions with minimal insolation (approximately 2,300–2,400 h per year) – Akmola and North Kazakhstan regions – demonstrate the highest suicide rates: approximately 28–29 per 100,000. At the opposite end of the scale is Kyzylorda region: with maximum insolation (approximately 3,000 h), it demonstrates the lowest suicide rate - approximately 11.5 per 100,000. In between, regions such as East Kazakhstan, Karaganda, Pavlodar, and West Kazakhstan regions occupy a middle position in both insolation and suicide rates. The southern regions – Zhambyl and Turkestan regions – with a higher number of sunny days record significantly lower rates.

The identified pattern is consistent with the hypothesis that insufficient sunlight plays a role in the development of depression and suicide risk, and also explains the persistent differences between the northern and southern regions of Kazakhstan. However, it should be noted that correlation does not imply causation – regional differences may be influenced by other factors, such as ethnic composition, level of urbanization, access to mental health care, and regional economic development.

## Discussion

This study presents the first integrated, nationally representative analysis of suicide mortality in Kazakhstan spanning a 13-year period (2012–2024), simultaneously characterizing temporal trends, demographic patterns, and regional distribution. The key findings are: (1) a 15.8% decline in absolute suicides against a backdrop of 19.3% population growth; (2) a pronounced male predominance (M/*F* = 3.8:1) across all age groups; (3) markedly different age-specific risk profiles by sex, with men exhibiting a bimodal trajectory; (4) exceptionally high regional variability with a clear north-to-south gradient; and (5) regional population aging as the strongest demographic ecological correlate, and annual sunshine duration as the strongest environmental ecological correlate in the regression model.

The observed 15.8% decline in absolute suicide cases between 2012 and 2024, occurring alongside a 19.3% increase in population, represents a meaningful reduction in suicide burden that exceeds what raw case counts alone would suggest. This trajectory is broadly consistent with global trends documented in the WHO Global Health Estimates, which reported declining age-standardized suicide rates in many countries over the past two decades (1–8). However, Kazakhstan’s rate at the end of the study period (SR = 17.8; ASSR = 18.2) remains more than 65% above the WHO global average, indicating that despite progress, suicide remains a disproportionate cause of premature mortality.

The unexpected 14.5% decrease in suicide cases recorded in 2020, followed by a 19.9% rebound in 2021, runs counter to patterns reported in several high-income countries during the COVID-19 pandemic, where rates either increased or remained stable (1–8). This observation is consistent with a broader literature suggesting that, in some contexts, the early pandemic period was associated with a temporary reduction in suicide mortality – a phenomenon attributed to increased social cohesion, government economic support measures, and the so-called “pulling together” effect that can occur during acute collective crises (2–5). The subsequent rebound in 2021 likely reflects delayed effects of pandemic-related economic disruption and mental health deterioration. However, a formal cross-national statistical comparison was not conducted in this study, and this observation should be interpreted with caution.

The male-to-female ratio of 3.8:1 in Kazakhstan is broadly consistent with patterns documented across the former Soviet space and in high-income countries, where male rates are typically two to four times higher than female rates (1, 7, 9, 10). A national analysis of 23,001 suicide deaths in Turkiye between 2018 and 2023 similarly found male rates to be approximately three times higher than female rates throughout the study period, with economic problems being six times more frequent among male victims than females – underscoring the role of gender-specific socioeconomic pressures in suicide risk across diverse cultural and socioeconomic settings (11). Several plausible explanations for this disparity have been proposed in the international literature, including men’s greater use of highly lethal methods, lower rates of help-seeking and mental health service utilization, and the psychological consequences of culturally entrenched expectations around the male breadwinner role (12–15). In Kazakhstan, the predominance of hanging (78.9%) and the high proportion committed while sober (80.8%) suggest that a substantial proportion of suicides may reflect chronic hopelessness and deliberate intent rather than impulsive behavior in the context of acute intoxication.

It is important to note that none of these mechanistic explanations were directly measured in the present study, and they should therefore be interpreted as plausible hypotheses grounded in the existing international literature rather than as findings supported by our data. The cited references (12–15) are not Kazakhstan-specific. Direct empirical testing of these hypotheses within Kazakhstan remains a priority for future research.

The age distribution of suicide risk differed markedly between men and women in Kazakhstan (16–19). Men exhibited a bimodal risk trajectory with distinct peaks at 25–29 years (SR = 38.6) and 45–54 years (SR = 49.9), reaching a maximum in the 75 + group (SR = 51.3). This pattern – an early adult peak corresponding to the transition into independent life, followed by a mid-life peak associated with occupational stress, economic pressure, and family responsibilities – is broadly consistent with findings from Turkiye, where suicides were most frequent among individuals aged 20–39, and where economic problems and illnesses were the leading documented causes in the 31–60 age group (11). The age-stratified distribution of suicide causes reported in that study – with familial problems dominating in younger age groups, economic difficulties peaking in middle age, and illness-related causes increasing in older age – maps closely onto the age-specific risk trajectory we document for Kazakhstani men.

In contrast, women in Kazakhstan demonstrated a single prominent early peak in the 18–24 age group (SR = 10.6), followed by a relatively stable risk trajectory until rates rose again in older age. This pattern may reflect the particular vulnerability of young women to relational stressors, early marriage pressures, and limited autonomy in some regions of Kazakhstan, though direct data on these factors were not available. The notably lower suicide rates among women throughout the working-age years – relative to men – likely reflects, at least in part, women’s greater propensity to seek mental health support, a well-documented sex difference in help-seeking behavior (12). The substantially higher proportion of female suicides occurring in the 5–17 and 75 + age groups relative to men warrants targeted attention in prevention programming.

Hanging accounted for 78.9% of all suicides – a proportion that is substantially higher than in most high-income countries. The ecological correlates of hanging as a method – lower urbanization and lower income levels (Table 1) – suggest that method availability and rural social norms may shape method choice (20). The negative correlation between urbanization and hanging (*r* = −0.62) indicates that in cities, where other means may be more accessible and awareness of alternatives greater, the dominance of hanging is attenuated. This has direct prevention implications: means restriction (e.g., safe storage messaging, physical barriers) may be particularly impactful in rural, lower-income regions where hanging is overwhelmingly predominant.

The high proportion of suicides committed while sober (80.8%) is also noteworthy (21). This distinguishes Kazakhstan’s suicide profile from the pattern in some other post-Soviet states where acute alcohol intoxication plays a more prominent co-occurring role. It suggests that a substantial proportion of completed suicides in Kazakhstan may reflect chronic hopelessness and deliberate intent rather than impulsive behavior in the context of acute intoxication – a distinction with important implications for the design of prevention interventions.

The regional distribution of suicide mortality in Kazakhstan revealed a pronounced north-to-south gradient, with rates in the Akmola and North Kazakhstan regions (>28 per 100,000) more than twice those recorded in the Kyzylorda and Mangystau regions (<12 per 100,000). This pattern has been consistently documented in several countries with similar climatic gradients, including France, Finland, Russia, the United States, and Thailand (22–30). In France, the Bretagne region recorded the highest suicide rate (24.7 per 100,000), while southern regions such as Île-de-France showed substantially lower rates (25). In Finland, the northern and eastern regions consistently show the highest male suicide rates (>35 per 100,000), with adolescent male firearm suicide rates in the north nearly three times those of the south (24). In South Korea, peripheral rural regions rather than strictly northern ones bear the highest burden, reflecting a rural–urban divide that partially parallels Kazakhstan’s urban–rural gradient (31–34).

Among the demographic ecological correlates examined, the regional average population age (*r* = 0.84) and aging index (*r* = 0.84) showed the strongest associations with regional suicide rates. An even stronger association was observed for the composite interaction term (*r* = 0.89), though this should be interpreted with caution as noted above. These correlates are stronger than the insolation correlation (ρ = −0.784), indicating that demographic restructuring – particularly population aging concentrated in northern regions – is the primary structural driver of regional variability, with insolation emerging as a significant correlate in the model specifically testing environmental and economic factors.

In the multiple linear regression model, annual sunshine duration was significantly and inversely associated with regional suicide rates (*β* = −0.814; 95% CI: −1.221 to −0.407; *p* = 0.001), while average regional salary was not (*β* = −0.032; *p* = 0.864). This finding is consistent with a growing body of ecological evidence linking lower solar insolation to higher population-level suicide rates, documented in Italy, Japan, and several European countries (35–41). A biological pathway linking reduced solar insolation to serotonin dysregulation, circadian disruption, and vitamin D deficiency – and through these to depressive symptoms and elevated suicide risk – has been proposed and partially supported in the existing literature (35–41). An autopsy-based study of 50 confirmed suicide deaths in Artvin, northeastern Turkiye, found that 73.5% of decedents had at least one chronic systemic disease and 46.9% had a documented psychiatric disorder or history of psychiatric medication use (42). However, individual-level data on sunlight exposure, depression, vitamin D status, or psychiatric diagnoses were not available in this study, and the proposed mechanism cannot be evaluated with the aggregate regional data used here. The observed association between regional sunshine hours and regional suicide rates is ecological in nature and should not be extrapolated to individual-level causal inference.

Crucially, this association should be interpreted strictly as an ecological correlate of regional suicide mortality and not as a causal or explanatory factor. The regression model included only two variables – insolation and salary – and stronger demographic correlates (average population age, aging index) identified in Table 1 were not incorporated. It is therefore possible that the observed insolation association partially reflects confounding by the regional demographic structure: northern regions of Kazakhstan are simultaneously older, more male-skewed, more rural, and less sunny than southern regions. Disentangling the independent contribution of insolation from that of demographic factors will require a multivariate model incorporating all relevant covariates.

Regional heterogeneity in the M/F ratio (range: 2.6–5.4) adds another layer of complexity. The most balanced gender ratios were observed in the cities of Astana (2.6) and Almaty (2.7), while the highest were in Zhetysu (5.4) and East Kazakhstan (5.1). This pattern likely reflects several plausible mechanisms proposed in the international literature: lower mental health help-seeking among rural men (12), greater occupational exposure to economic shocks in agriculture- and industry-dominated northern regions (13), and the psychological consequences of breadwinner role loss among men with strong gender-traditional identities (14). However, none of these factors were directly measured in the present study, and the cited evidence is not Kazakhstan-specific. Direct empirical testing of these hypotheses within Kazakhstan remains a priority for future research.

Urban women are generally more willing to seek mental health care, while men – especially in rural areas – are less likely to do so. This differential in help-seeking behavior may partly explain the lower M/F ratios observed in urban settings.

Comparison with recently published data from Turkiye provides a useful external reference point. In contrast to Kazakhstan, Turkiye’s overall suicide rate (4.73 per 100,000) is substantially lower than both the global average and Kazakhstan’s national rate – a difference potentially attributable to religious and cultural factors, including the social stigma attached to suicidal behavior in predominantly Muslim societies (11). Nevertheless, several structural patterns are shared across both countries: the male predominance (approximately 3:1), the concentration of economic problems as a documented cause of suicide particularly among men (six times more frequent in males in Turkiye), and the predominance of hanging as the primary method (47.2% in Turkiye vs. 78.9% in Kazakhstan). Furthermore, trend analysis in Turkiye indicated a significant annual increase of 3.6% in total suicides (IRR = 1.036; *p* < 0.0001), in contrast to the declining trend observed in Kazakhstan – suggesting diverging trajectories in two neighboring countries with some shared cultural features (11). An autopsy-based study of confirmed suicide deaths in Artvin, northeastern Turkiye, found that 73.5% of decedents had at least one chronic systemic disease and 46.9% had a documented psychiatric disorder or history of psychiatric medication use – frequently both concurrently (42). This underscores a consistent finding in the international forensic literature: population-level demographic and environmental factors coexist with individual clinical and psychiatric vulnerabilities, the latter of which cannot be captured in aggregated mortality datasets such as those used in the present study.

The ecological design of the regional analyses is susceptible to residual confounding by variables not included in the models. Regional differences in ethnic composition, religion, alcohol consumption, cultural attitudes toward help-seeking, and the density of psychiatric services may all contribute to the observed patterns independently of the demographic and environmental variables measured. The associations reported in Table 1 and the regression model should therefore be treated as hypothesis-generating rather than as evidence of independent causal effects. The multiple correlations in Table 1 were exploratory; a Benjamini-Hochberg FDR correction was applied and all significant associations remained significant after adjustment.

The findings of this study have direct implications for suicide prevention policy in Kazakhstan. Four priority areas can be identified.

First, the exceptional concentration of suicide mortality in northern Kazakhstan – where rates in Akmola and North Kazakhstan exceed the national average by more than 50% – calls for region-targeted resource allocation. Prevention efforts and mental health infrastructure should be prioritized in these areas, including mobile psychiatric teams, crisis hotlines accessible to rural populations, and primary care integration of mental health screening.

Second, men in the 45–54 age group represent a particularly high-risk demographic that has received limited targeted attention in Kazakhstan’s prevention framework. Interventions addressing financial distress, unemployment support, and male-specific help-seeking promotion may be especially valuable in northern and rural regions.

Third, the consistently lower suicide rates in Kazakhstan’s three major cities suggest that the urban environment may offer protective effects through greater access to mental health services, economic opportunity, and social integration. Systematic study of which specific urban factors account for this protective effect could yield transferable lessons for rural prevention programs.

Fourth, the high proportion of suicides committed by hanging (78.9%) points to means restriction as a potentially high-impact prevention lever. Evidence from other countries suggests that reducing access to lethal means can produce meaningful reductions in suicide mortality, particularly where hanging predominates. Kazakhstan’s national prevention strategy should consider targeted education and environmental modifications as a complement to clinical and social interventions.

The finding that age-standardized rates remain more than 65% above the WHO global average despite meaningful overall declines emphasizes that Kazakhstan’s current prevention infrastructure has achieved progress but remains insufficient to close the gap with global benchmarks.

## Conclusion

The analysis showed that SR patterns in Kazakhstan are generally consistent with global epidemiological trends, particularly regarding the high male-to-female ratio, more complex age-related risk dynamics for men, and the highest rates observed in individuals over 75. At the same time, the results highlighted that Kazakhstan’s regions exhibit exceptionally high variability in SRs, with a pronounced north-to-south gradient. Regional demographic restructuring – particularly population aging and the concentration of older men in northern regions – is the primary structural ecological correlate of regional suicide variability, with the aging index showing the strongest association (*r* = 0.84–0.89). Annual sunshine duration emerged as a significant ecological correlate in the regression model specifically designed to test environmental and economic factors (*β* = −0.814; *p* = 0.001), but this association may not be fully independent of the underlying demographic structure. Future multivariate analyses should simultaneously incorporate demographic, environmental, and socioeconomic variables to estimate their relative independent contributions.

Given the strong ecological association between regional aging indices and suicide rates observed in the 2023–2024 cross-sectional data, longitudinal monitoring of demographic trends in high-risk northern regions is warranted. However, this study did not conduct demographic projection or time-series forecasting analyses, and projections of future national suicide rates based on aging trends fall outside the scope of the present investigation. This analysis provides an evidence base for developing targeted preventive measures.

## Data Availability

The raw data supporting the conclusions of this article will be made available by the authors, without undue reservation.
